# Emotional reactivity and perspective-taking in individuals with and without severe depressive symptoms

**DOI:** 10.1038/s41598-018-25708-x

**Published:** 2018-05-16

**Authors:** Constance Imbault, Victor Kuperman

**Affiliations:** Department of Linguistics and Languages, McMaster University, Hamilton, Ontario, L8S 4L8 Canada

## Abstract

The perspective-taking ability to imagine another person’s feelings and thoughts is paramount for successful communication. This study pursued two questions regarding the link between perspective-taking and depressive symptomatology in a task where participants provided responses to words ranging in their positivity. First, we examined in a between-participants experimental manipulation how the presence of depressive symptoms influenced participants’ emotional reactivity. Second, we measured within-participants, how their responses change as a function of the perspective they are assigned to take, that of a depressed or a non-depressed person. Our main interest is in the interaction of the two effects: we examine how one’s emotional state determines the ability to engender someone else’s responses. Our central finding is that depressive symptoms lead to emotional insensitivity, i.e., weaker responses to extremely positive and negative words. Furthermore, depressive symptoms come with a much weaker ability to take a non-depressed perspective. Finally, non-depressed participants demonstrated an excellent ability to mimic the blunt affect of depression when responding for the other group, suggesting that the outlook of a depressed individual is available to people throughout the range of depressive symptomatology. We discuss the implications of these findings for quantifying emotional reactivity during depression, as well as the diagnosis and prognosis of depression.

## Introduction

The ability to take the perspective of another person, i.e., to imagine that person’s feelings and thoughts, is at the core of human social interaction and is the glue for a functional society^1,2^. A central question we ask in this paper is whether the perspective-taking ability undergoes any transformation in depression. This question requires knowledge of how well-developed perspective-taking is in non-depressed individuals and how the presence of depressive symptoms affects it. Specifically, we examine two novel questions: (i) whether people with depressive symptoms can imagine emotional states of non-depressed people and (ii) how accurately can non-depressed people mimic the emotional states of depression. Our choice of depression for pursuing question (i) has several reasons. First, the very definition of this mood disorder cites “hopelessness, anhedonia, loss of interest” and other markers of affective impairment^3^. Thus, one’s emotional state in depression tends to differ greatly from a non-depressed one. The literature also reports that depression comes with a weakened Theory of Mind and thus a weaker understanding of others’ emotions^2,4,5^. This is possibly due to increased self-focused attention^6^, an obvious obstacle to taking another perspective. Whatever the cause, the deficit in perspective-taking is clinically important, because it has been argued to lead to a feeling of interpersonal isolation and loneliness^1,7^, exacerbating depression and social anxiety^8–10^.

Despite the monumental literature of depression and affect, there has been little to no direct research on perspective-taking in this literature, as opposed to related concepts of empathy and Theory of Mind^4,5,11–13^. In this paper, we make a distinction between perspective-taking and Theory of Mind. We define Theory of Mind as the ability to understand that others have their own mental states that are different from our own^2^. Perspective-taking, on the other hand, is the ability to put yourself into someone else’s shoes; to understand and perceive *how* others feel^14^. Though these terms are related and often confused, we construe them as different constructs. The keyword search with “perspective taking” and “depression” retrieved only 42 PUBMED citations on September 5^th^, 2017, none of which directly address this relationship, but see^15^. Intriguingly, the opposite is also true: little is known about whether a non-depressed person can construe the point of view of a person with depression, i.e., our question (ii). Yet there are indications that this ability is important to the prognosis of depression. Increased perceived social support and understanding are negatively correlated with depression severity^16^ and protect against postpartum depression^17^. Thus, our second focus is on whether non-depressed individuals can mimic the emotional state of depression.

Our study examines individuals’ responses to a broad range of emotion-laden linguistic stimuli, while those individuals take on a perspective of a person with and without depression. This task is administered within-subjects, giving insight into how strongly the change in perspective influences the magnitude and polarity of emotional responses. We manipulate the required perspective and select individuals with varying severity of depressive symptoms. Specifically, we quantify how the severity of one’s depressive symptoms (as measured via a clinical self-report assessment) modulates one’s ability to take another perspective. There are at least two ways to approach our questions. One possibility is to ask participants to self-identify as depressed or non-depressed, and then instruct them to complete the task for their own group and the opposite group. This approach would afford an insight into a person’s own emotional reactivity, but it would also come with a cost. Specifically, the experimental demand of categorical self-identification can elicit cognitive and social response biases (including the in- and out-group stereotyping)^18^ when taking their own purported perspective and the opposite perspective. For these reasons, we took a different approach: we did not ask participants to identify their depression status, and we instructed all of them to complete a task from the perspective of a depressed and a non-depressed person. One aspect of their behaviour that we cannot measure using this approach is the knowledge of how participants would have behaved with their own perspective. We do, however, gain insight into their perspective-taking abilities, operationalized as a difference between two perspectives that they have to take. This insight is the focus of our study; we leave the exploration of the first approach to future research.

In what follows we formulate our hypotheses and outline an experimental task that enables these measurements.

First, if individuals can take on the perspective of someone with depression, they are expected to show affective behaviour similar to that found in depression. What does this behaviour look like? People who have depression experience a pessimism bias and predict negative outcomes for their future^19^, whereas people without depression are more optimistic^20^. But the exact characteristics of affective behaviour in depression are unclear. One of the most common symptoms is anhedonia, or the loss of pleasure: it leads to reduced positive affect and weaker responses to positive stimuli, known as positive attenuation^21,22^. Other literature reports that those with depression have blunted emotion across the entire range, emotion context insensitivity, and their perception of the world is neither positive nor negative, but neutral^23,24^. Behaviourally, this surfaces as lethargy, fatigue, and lack of influence from external emotions. In other words, a “depressed” perspective might arguably cause responders to give weaker responses to positive words only (replicating positive attenuation) or to the entire word range (replicating emotion context insensitivity). Conversely, an assumed “non-depressed” perspective is expected to lead to a broader, unconstrained range of emotional responses.

Second, in line with the literature on traits similar to perspective-taking, such as Theory of Mind, we expect the responder’s severity of depressive symptoms to modulate their perspective-taking ability. An oft-cited reduction in the Theory of Mind performance among depressed individuals, as well as a relatively pessimistic outlook associated with hopelessness suggest that depressive symptoms may come with inability to put oneself into another’s shoes^4,5,13,19,25,26^. Thus, we predict that, while taking different perspectives, an average individual with depressive symptoms will change their emotional responses to words to a lesser degree than an average individual without these symptoms.

**Figure 1:**
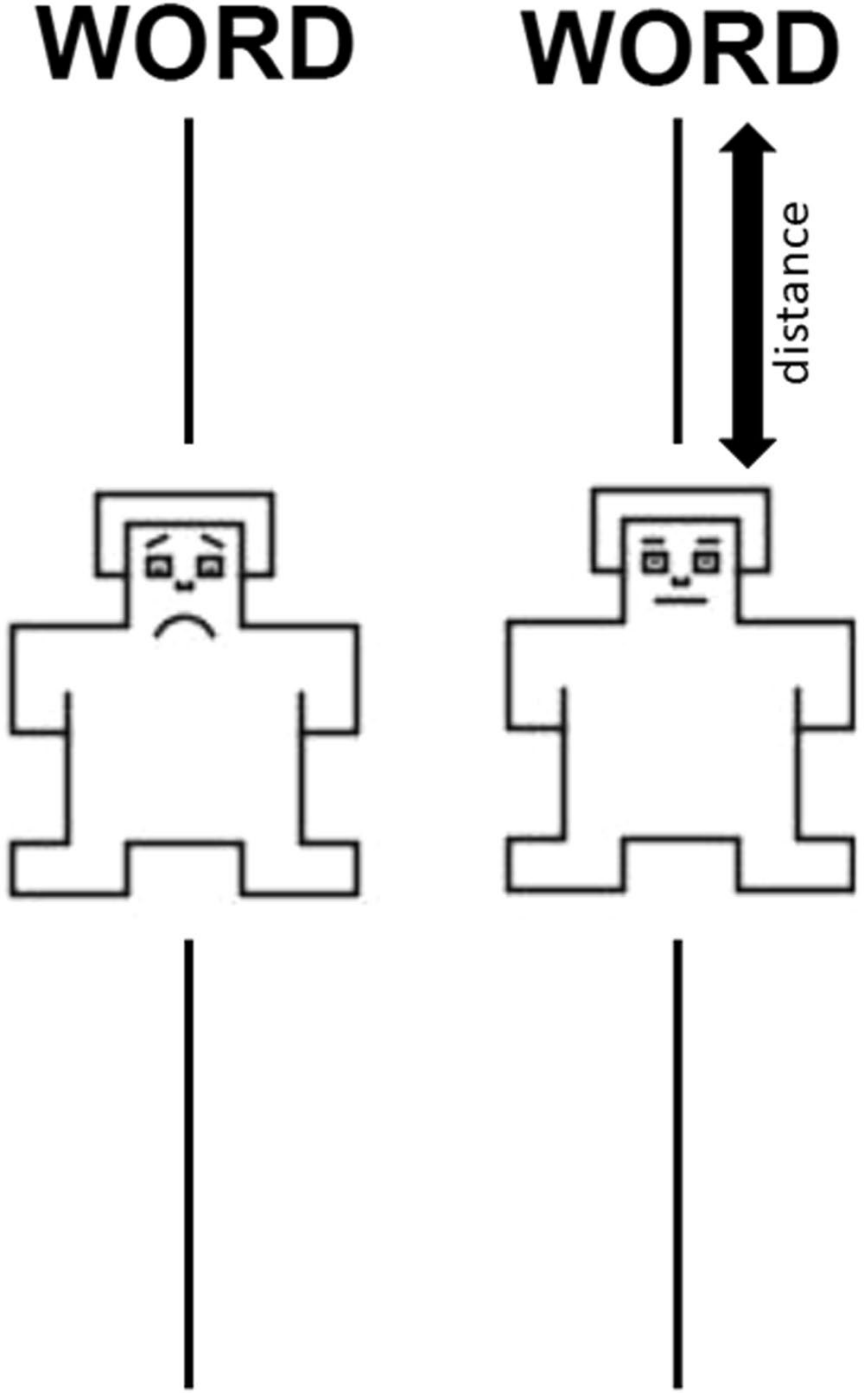
Illustration of the two conditions in the slider task with a sad (left) and neutral (right) manikins and how the distance to the word is calculated. Participants only see one manikin at any given time.

An experimental paradigm that enables us to pursue these hypotheses within-subjects is that of a slider task^27^. The task consists of a scale and a humanoid manikin. The manikin is placed in the center of a (typically vertical) scale on a computer screen and can either represent the participant or someone else. Additionally, a word appears at the top or the bottom of the screen. Participants are instructed to move the manikin as close to, or as far away from, the word as they would prefer to be: they can be instructed to convey their own judgment or that of another person’s perspective. The distance the manikin is from the word represents the affective rating of that word by the participant (smaller distance is higher valence; see Fig. 1). Warriner *et al*.^27^ have found that the affective ratings from this task, when the referent was the participant, strongly correlated with affective ratings from a Likert scale, while^28^ have demonstrated that the slider task is a reliable measure of valence for words both within and across individuals. The benefit of the slider method, compared to the ratings method, is that we can easily change the referent for the affective ratings, asking people to rate the emotion of words for someone who is not themselves. In the present task, all participants were asked to respond on a slider scale to a selection of words representing the entire spectrum of positivity and negativity, taking on the perspective of either a depressed or a non-depressed person. The outcomes allow for an easy quantification of the influences that perspective-taking and the presence or absence of depressive symptoms have on affective behaviour.

## Results and Discussion

The initial raw data set consisted of 13,458 trials. Fifteen participants completed the task (at least partially) twice; we removed their second pass from analyses (2,808 trials), resulting in 10,650 trials. An additional 300 trials (2 participants) were removed from analysis due participants not completing the QIDS, resulting in 10,350 trials. We then, post-hoc, removed participants who fell outside of our extreme-groups QIDS criteria, outlined above. The resulting data pool contained 6,877 trials from 47 participants.

The Basic Empathy Scale can be broken down in two sub-scales; cognitive empathy and affective empathy. Overall empathy scores for the non-depressed group, which ranged from 59 to 95 (*M* = 75.923, *SD* = 10.103), were not significantly different from the depressed group, which ranged from 60 to 98 (*M* = 80.476, *SD* = 10.196) (*t* = −1.528, *p* = 0.134). Additionally, cognitive empathy scores for the non-depressed group, which ranged from 28 to 43 (*M* = 37.54, *SD* = 4.12), were not significantly different (*t* = −0.089, *p* = 0.93) from the depressed group, which ranged from 26 to 45 (*M* = 37.67, *SD* = 5.46). Interestingly, the depressed group had higher affective empathy scores than the non-depressed group (depressed: range 30:53, *M* = 42.81, *SD* = 7.35; non-depressed: range 26:52; *M* = 38.38, *SD* = 7.25; *t* = −2.06, *p* < 0.05). All empathy scores were not found to influence individual performance in the slider task, nor did they enter into an interaction with any critical variable: they are not reported further. We thank an anonymous reviewer for suggesting this analysis.

**Figure 2:**
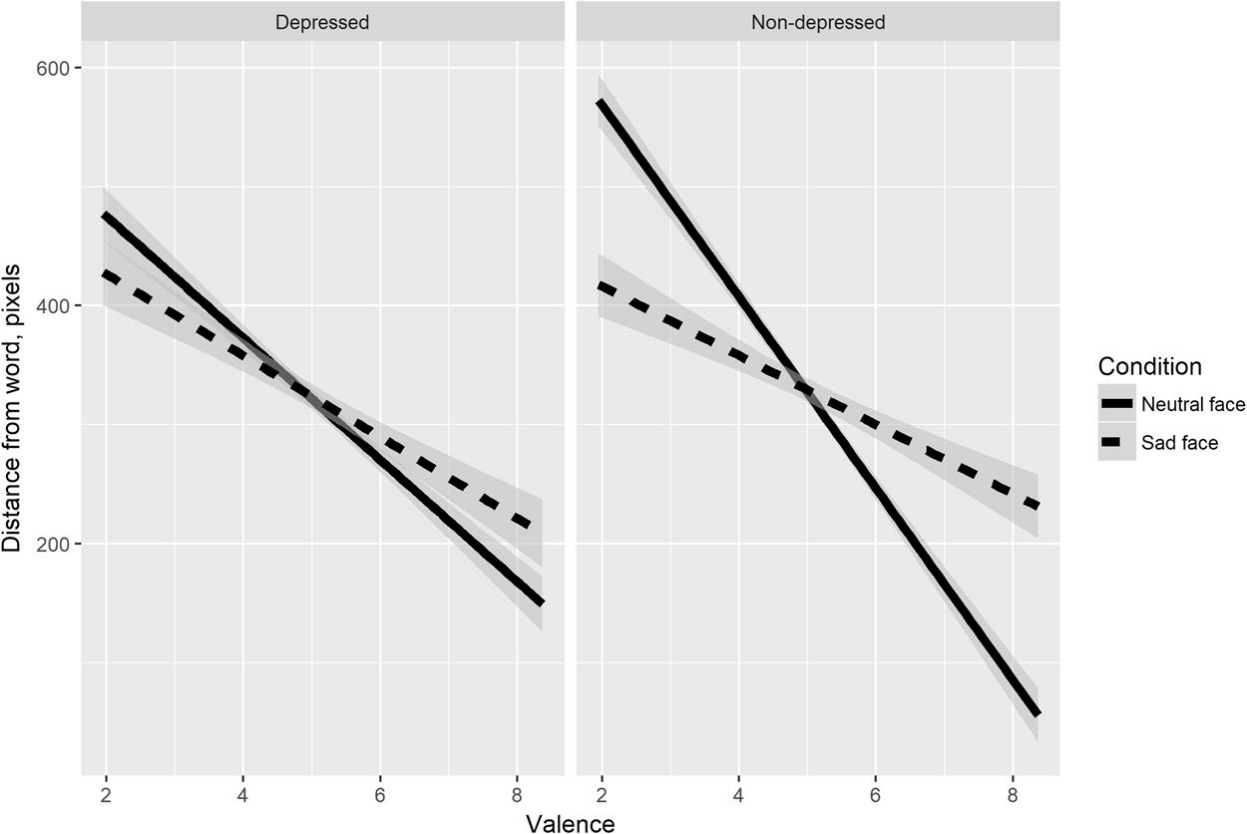
Distance from the word shown by depressed and non-depressed participants with both conditions (sad and neutral face).

A regression model with the three-way interaction of Face × Group × Valence quantifies changes in affective behaviour (manikin’s distance to target word) caused by the assumed perspective, word valence and the severity of depressive symptoms, see Table 1. Figure 2 visualizes the response patterns by presenting the effect of valence on distance for a depressed group (left panel) and a non-depressed group (right panel) and for a sad and neutral manikin (dashed and solid lines respectively). The plot is based on linear fits to raw data: visual inspection of a plot with model predictions reveal that they are a close fit to Fig. 2. In what follows, we describe patterns of interest based either on the model with a three-way interaction in Table 1 or the models that highlight specific contrasts by using two-way interactions nested in that bigger model (not shown).

**Table 1:** Linear mixed-effects model fitted to the distance of the manikin from the word.

Predictor	Estimate	*SE*	*t*-value	*p* value
Intercept	322.309	8.753	36.823	<0.001
Valence	−33.803	12.319	−2.744	0.008
Non-depressed Group	2.730	11.185	0.244	0.808
“Neutral” Face	−9.208	6.345	−1.451	0.147
Valence: Non-depressed Group	5.508	16.297	0.338	0.737
Valence: “Neutral” Face	−18.036	5.104	−3.534	<0.001
Non-depressed Group: “Neutral” Face	0.242	8.637	0.028	0.977
Valence: Non-depressed Group: “Neutral” Face	−35.487	6.975	−5.088	<0.001

N = 6858. SD of random by-word intercepts = 33.55, SD of random by-subject intercepts = 32.08, SD of by-subject random slopes of valence = −0.21, SD of residual = 173.30. Reference levels are “depressed” for Group, and “Sad” for Face.

In all conditions, greater word valence came with a shorter distance between the manikin and the word. In line with all prior reports^27,28^, positive emotion is associated with an approach behaviour. However, marked differences were observed both across groups and across experimental conditions in how strongly the range of positivity influenced responses to words. The steepest slope (and the stronger effect of valence and range of responses) was found in the non-depressed group taking a neutral perspective (*B* = −79.58, which is a measure of change in pixels per 1 unit of valence). The respective slope values for depressed participants with neutral manikin, non-depressed participants with sad manikin and depressed participants with sad manikin were *B* = −51.44, −26.23 and −33.46.

These data enable us to answer the questions posited in the Introduction. Our first analysis compares the change in perspective, arising from completing the task with a sad face versus a neutral face (i.e., mimicking a non-depressed versus a depressed perspective). Both groups changed their behaviour in a way that showed a blunter affect with a sad, depressed perspective (dashed lines had flatter slopes than solid ones in both panels). Yet in the non-depressed group the change in perspective had a much stronger effect on distance than in the depressed group (non-depressed: B = 52.987, SE = 4.637, t = 11.428, p < 0.001; depressed: B = 18.309, SE = 5.153, t = −3.553, p < 0.001). This cross-group difference between conditions was significant, as the three-way Face × Group × Valence interaction showed (B = −35.487, SE = 6.975, t = −5.088, p < 0.001). Taken together, these findings indicate that all participants are capable of perspective-taking, yet this ability is weakened in people with depressive symptoms.

How good were participants in changing their perspective? Individuals in the non-depressed group were extremely accurate in approximating the behaviour of individuals with depressive symptoms when both groups were moving the sad manikin (dashed lines across panels in Fig. 2). There was no reliable contrast in the valence slopes for these two groups when operating the sad manikin (B = 5.686, SE = 16.369, t = 0.347, p = 0.730). However, the opposite was not true. Individuals with depressive symptoms were unable to change their perspective and replicate the wide range of emotional responses characteristic of non-depressed individuals when both groups moved the neutral manikin. The difference between the two groups’ slopes in the neutral-face condition was marginally significant (B = 29.889, SE = 16.241, t = 1.840, p = 0.07), with the depressed group’s slope being 29.89 pixels (or 4.5% of the available distance scale) flatter than the non-depressed group per unit of valence (solid lines across panels in Fig. 2). Across the entire range of valence, this difference equates to 29.57% of the available scale.

Finally, the patterns demonstrate that either depressive symptoms or a depressed perspective have impact on the entire range of valence, and not specifically positive or negative stimuli. A hypothetical regression line that would show a perfect symmetry between impacts on positive and negative words is predicted to have an intercept of 325 pixels (the middle of the slider scale) at the mean value of the valence range. In fact, all regression lines were nearly perfectly centered: all intercepts were within 10 pixels of the mid-point of the slider scale, see Fig. 2 and relevant contrasts in Table 1. This finding sheds light on a long-standing debate of whether the pessimistic outlook only influences the positive side of the emotional spectrum, its negative side, or the entire spectrum: the latter is true.

## General Discussion

This study pursued two novel questions regarding emotional states of depressed and non-depressed individuals and their respective abilities to take each other’s perspective. First, we quantified between participants how the presence or absence of depressive symptoms influenced the magnitude of their responses to words representing an entire range from very negative to very positive. Second, we measured within-participants how their responses change as a function of the perspective they are assigned to take, that of a depressed or a non-depressed person. Our main interest is in the interaction of the two effects: we examine how one’s emotional state determines the ability to imagine someone else’s responses.

Unsurprisingly, individuals with depressive symptoms respond differently to emotional stimuli than those without depression. In line with previous literature^21–24^, when the participants with depressive symptoms operated the sad manikin, they did not use the entire range of the slider scale, but instead remained relatively neutral to all levels of valence. One of our questions concerned whether blunted emotion was specific to positive attenuation^21,22^ and thus restricted to anhedonia, or the entire scale, expressed as emotion context insensitivity^23,24^. Our data offer a clear demonstration of the latter hypothesis, in support of a notion that an across-the-board flattening of affect is symptomatic of depression^3^. On the other hand, and consistent with previous research with the slider paradigm, participants without depression were strongly effected by valence and used the entire range of the slider scale in response to valence when moving a neutral manikin^27,28^.

A more intriguing new finding is the effect of depression on perspective-taking abilities. The findings that those with depression have heightened empathy and lowered Theory of Mind are well established in the literature^4,5,11,12,26,29^, but there has been little to no research on how depression would impact perspective taking abilities. By manipulating the slider paradigm, i.e., by asking those with depression to behave like people without depression, we were able to quantify this impact. The comparison was achieved by the analysis of the slider task performance across depressed and non-depressed groups when operating a neutral manikin. When answering for another group, the participants with depressive symptoms did not match the large effect that valence had on distance for that (non-depressed) group. Instead they responded with a more shallow slope, i.e., distance from the word was less influenced by valence. These responses in the depressed group were similar to those they produced when operating a sad manikin. We take this to mean that our participants with depression had trouble with taking the perspective of a person without depression. An alternative interpretation of these results is that participants with depressive symptoms have overall smaller emotional reactivity, and their observed inability to take on other perspective is just an inability to expand their emotional range. Although it cannot be ruled out given the data, the experimental design does not warrant this interpretation. Perspective-taking is purely a mental exercise; we do not ask participants to *feel* these emotions, rather we ask them to speculate how another group feels these emotions. Thus, their own reduced emotional reactivity per se is not expected to prevent participants with depressive symptoms from hypothesizing what emotional responses would be plausible under a different perspective. Thus, arguably, an inability to take on another perspective is at least partly independent from one’s range of emotional reactivity.

Our findings are consistent with such theoretical accounts as the Hopelessness Theory of Depression^25^ and the pessimism bias^19^. An inability to understand what it feels like to not be depressed is a harrowing finding, and leads into a “no light at the end of the tunnel” feeling of hopelessness. Autonomous motivation, i.e. “I want to get better”, is a strong predictor of the outcome of depression, resulting in higher remission and lower depression severity^30^. Our findings indicate that a critical component for this motivation is missing in the outlook of a person with depressive symptoms: namely, an understanding of what being better feels like.

Another new finding is that non-depressed participants were able to accurately adopt the perspective of the feeling of depression. When these participants moved the sad manikin, they accurately approximated the much weaker effect of valence on the distance from the words. The observation that emotional states linked to depressive symptoms are accessible to the majority of the population is valuable for the prognosis of depression. As shown in studies of this mood disorder, having a strong social network and feeling understood leads to faster recovery^9,10,16,17^.

To our knowledge, this is the first time when perspective-taking ability has been directly tested within subjects and in the context of depressive symptomatology. We observed in two groups with and without severe depressive symptoms highly distinct patterns of affective behaviour, which were contingent on both their own symptomatology and who they represented in the task. Depressive symptoms restricted an emotional range to the point where a “normal” perspective appeared unattainable. Conversely, depressive mood was an easy target for people who were not in that emotional state. This set of findings is noteworthy because of its potential implications for diagnostics and therapy of depression. It is also notable because it was obtained with a very simple online experimental tool in a non-invasive way and yet gave rise to strong and statistically reliable differences between groups and within individuals. The new slider method that we introduced offers easy quantification of every aspect of participants’ behavior and the independent and interactive contributions of all manipulated factors. As such, this tool enables easy replication in similar non-clinical cohorts and, importantly, a much-needed further expansion over clinical populations. The slider task can also quantify perspective-taking abilities in many groups, including those varying in gender, age, cultural background, or indeed their clinical status.

## Methods

### Participants

We recruited participants from a pool independently created by the BrainTrain project^31^ using the crowd-sourcing platform Amazon Mechanical Turk (www.mturk.com). This study was approved by the McMaster Research Ethics Board, protocol 2011 165, informed consent was obtained from all participants explaining any risks involved, and all methods were carried out in accordance with relevant guidelines and regulations. 71 participants from the pool agreed to take part in our study and were compensated monetarily. All but 2 participants had completed QIDS-SR prior to being recruited for this experiment (The Quick Inventory of Depressive Symptomatology; a self-report, 16-item questionnaire about the symptoms of depression^32^); the data for two participants were removed from analysis. We adopted the clinical cut-offs of the QIDS-SR scale, and defined a non-depressed group as one with QIDS-SR score < 6 and a group with moderate to severe symptoms of depression as one with QIDS-SE score ≥ 11. We removed 22 participants that did not meet the cut-offs of our extreme groups design.

The remaining 47 participants (23 female) ranged in age from 19 to 72 (*M* = 34.74, *SD* = 13.24). The 26 participants (9 female) in the “non-depressed” group ranged in age from 19 to 72 (*M* = 37.27, *SD* = 15.61). Their QIDS-SR scores ranged from 0 to 5 (*M* = 2.385, *SD* = 1.65). The 21 participants (14 female) in the “depressed” group ranged in age from 20 to 50 (*M* = 31.62, *SD* = 8.94). Their QIDS-SR scores ranged from 11 to 22 (*M* = 14.10, *SD* = 2.96).

It is important to note that our participants were selected based on their QIDS-SR score, which is a self-reported questionnaire about the *symptoms* of depression. We have no information on whether our participants actually had clinical depression. Thus, our group labels “depressed” and “non-depressed” refer to the *symptoms* exhibited at the time of self-report, and not necessarily to the presence of the mood disorder. Participants were not asked whether they self-identified with having a depression (see motivation above). However, we made a simplifying assumption that the affective behaviour shown by participants with depressive symptoms when taking a “sad, depressed” perspective is representative of their own behaviour, much like the behaviour shown by non-depressed individuals with a neutral manikin is indicative of their own behaviour.

### Affective Stimuli

A set of 150 words were selected to represent an entire range of psychological valence (positivity and negativity) and arousal. The norms of valence and arousal are available in Warriner *et al*.’s^33^ and are evaluated on a scale from 1 (sad or calm) to 9 (happy or excited). First, we chose monosyllabic words from Warriner *et al*.’s^33^ list of 13,763 words. These words were divided into 25 bins (5 quintiles of valence × 5 quintiles of arousal), and 6 words were randomly selected from each bin, guaranteeing a relatively uniform coverage of the affective range. The words had an average length of 4.34 characters (*SD* = 0.92). The mean frequency of occurrence in the 51 million-token corpus of subtitles to the US films and media SUBTLEX was 2780.53 (*SD* = 6338.43)^34^. Frequency was not correlated with arousal (${r}_{s}$ = 0.05, *p* = 0.51), but was correlated with valence (${r}_{s}$ = 0.31, *p* < 0.01). Valence and arousal were not correlated (${r}_{s}$ = −0.05, *p* = 0.54).

### Procedure

Participants completed the slider task on a web application that was implemented using jQuery and PHP. They used their own computers and were required to do so in Full Screen mode. Participants could use either a trackpad or a mouse to perform the experiment.

Prior to the start of the experiment, participants filled out demographic information (including age, sex, handedness, current US state and native languages). Immediately after, participants completed the Basic Empathy Scale^35^ which includes 20 questions on a 5-point Likert scale (“Strongly Disagree” to “Strongly Agree”) that measures both cognitive and affective empathy.

After completing the Basic Empathy Scale, participants read the instructions for the slider task: *“*[…] *you will see a word either at the top or the bottom with a vertical line below or above it*. *There will be a person in the centre of that line*. *The person represents either a neutral person or a sad*, *depressed person*. *You can move the person closer to or further away from the word*. *Position the person where you think they would prefer to be*”.

The “person” (manikin) was positioned in centre of the line, with a word at the top or the bottom of the screen (see Fig. 1). This task is an adaption of Warriner *et al*.^27^. Rather than asking participants to respond to the emotion of word for themselves, we asked the participants to respond as either a “sad, depressed” person, or a “neutral” person.

After reading the instructions, participants completed 5 practice trials with both types of manikin before continuing the experiment. The affective manikins were selected from Bradley & Lang^36^; the neutral manikin is the middle manikin from the valence scale, and the sad manikin is the lowest valence manikin from the valence scale. The emotion of the manikin was indicated at the beginning of each block and was confined to the shape of the mouth and the eyebrows (straight line for both in the neutral manikin, and raised eyebrows and a downturned mouth in the sad one, see Fig. 1). The manikin started in the centre position of the scale; 325 pixels away from the word. The participants were able to move the manikin as many times as they felt necessary before clicking the Submit button, but only the final position of the manikin was used in our data analysis. Every participant saw all 150 words, and word order was randomized within each block. Order of the blocks were counter balanced; half of the participants saw the sad manikin first, while the other half saw the neutral manikin first. All participants saw the same 75 words for the sad manikin and the same 75 words for the neutral manikin.

### Variables

The dependent variable of interest was the distance of the manikin from the word (measured in pixels) when the Submit button was pressed (labeled as Distance). The distance occupied a range of 648 pixels, 1 being closest to the word and 649 being furthest from the word.

One independent variable of interest is the word’s valence rating (Valence), which was retrieved from Warriner, Kuperman, & Brysbaert^33^. The valence ratings were the mean response of 20 raters given on a 1 (unhappy) to 9 (very happy) scale. An additional variable of interest was how empathetic the participants was, based on their Basic Empathy Scale score^35^. Another critical variable was Group, a between-subjects binary indicator of a group label (depressed or non-depressed) to which a participant belonged, based on the QIDS score^36^. Finally, a within-subjects binary indicator of the manikin type Face (sad vs neutral) marked the perspective that the participant was instructed to take. We expected the effects of interest to emerge in three or four-way interactions of these critical variables.

### Statistical Analyses

We used linear mixed-effects multiple regression models with participants and words as crossed random effects^37,38^, as implemented in package lmerTest version 2.0-33^39^ for R version 3.4.1^40,41^. This method enables a simultaneous exploration of multiple factors and covariates, while accounting for between-participants and between-items variance. Each model was initially fitted with random intercepts for words and participants and by-participant random slopes of valence, and trimmed down to only contain the random effects that significantly improve the model’s performance, as indicated by a series of likelihood ratio tests that compared a model with a given random effect and a model without this random effect. Using the same test in the backward elimination procedure, we removed all fixed effects that did not improve the model’s performance. To reduce the influence of outliers, the frequency estimates were (natural) log-transformed, as indicated by the Box-Cox power transformation test. After each fit, we excluded any data with residuals that fell 2.5 standard deviations away from the mean and refitted the trimmed models. Results are visualized with the help of the ggplot2 package^42^. Contrasts were coded with dummy coding.

### Data availability

The datasets analysed during the current study are available from the corresponding author (imbaulcl@mcmaster.ca) on request.
